# Case Report: A case report of myocardial fibrosis activation assessment after unstable angina using ^68^Ga-FAPI-04 PET/CT

**DOI:** 10.3389/fcvm.2024.1332307

**Published:** 2024-01-23

**Authors:** Mimi Jiang, Guolan Zhang, Le Li, Yuanyuan He, Guo Li, Jinmei Yu, Jian Feng, Xing Liu

**Affiliations:** Department of Cardiology, The Affiliated Hospital of Southwest Medical University, Luzhou, China

**Keywords:** unstable angina, myocardial fibrosis, ventricular remodeling, ^68^Ga-FAPI, PET-CT

## Abstract

Myocardial ischemia may induce myocardial fibrosis, a condition that progressively leads to ventricular remodeling, heightening the risk of heart failure. The timely detection of myocardial fibrosis is crucial for intervention and improved outcomes. ^68^Ga-FAPI-04 PET/CT shows promise in assessing fibroblast activation in patients with early myocardial infarction characterized by prolonged myocardial ischemia. However, there is a notable absence of data regarding patients with short-term myocardial ischemia, such as those experiencing unstable angina (UA). In this report, we evaluated a 49-year-old male with UA and severe stenosis in multiple coronary arteries using ^68^Ga-FAPI-04 PET/CT. The results demonstrated tracer-specific uptake (SUVmax = 4.6) in the left anterior descending artery (LAD) territory, consistent with myocardial anterior wall ischemia indicated by the electrocardiogram. Following vascular recanalization therapy and regular medication treatment, the patient remained free of angina recurrence. A subsequent review at 2 months revealed a significant reduction in myocardial tracer uptake (SUVmax = 1.8). This case illustrates the validity of ^68^Ga-FAPI-04 PET/CT in assessing the extent of early myocardial fibroblast activation in patients with UA. This approach offers valuable insights for early detection and visual evidence, providing information on disease progression and treatment response.

## Introduction

Unstable angina (UA) arises from a confluence of intricate factors that give rise to transient and reversible reductions in coronary blood flow. These contributing factors encompass vasoconstriction, transient platelet plugging, and transient thrombosis, collectively precipitating short-term myocardial ischemia (1). A subset of patients experiencing UA may undergo recurrent myocardial ischemia, potentially fostering pathological ventricular remodeling and an escalated risk of heart failure and arrhythmias (2, 3). Among these, fibrotic response plays a critical role in ventricular remodeling. In the context of myocardial ischemia-induced damage and inflammation, the release of TGF-β_1_ by inflammatory cells serves to activate fibroblasts to differentiate into collagen-secreting myofibroblasts. They secrete increased amounts of cytokines and TGF-β_1_ to regulate the deposition of matrix proteins, including fibronectin, type I and III collagen fibers, and proteoglycans. Excessive fibrosis and the persistence of active fibroblasts contribute to heightened left ventricular stiffness, thereby impacting diastolic or systolic function (4, 5). However, current myocardial fibrosis imaging methods commonly used in clinical practice, like CMR-obtained LGE and post-contrast myocardial T1 techniques, have limitations as they primarily measure extracellular expansion rather than fibrosis itself (6). Therefore, noninvasive imaging of activated fibroblasts could provide the information of myocardial fibrosis in the initial stage and unique opportunities to monitor therapeutic interventions that aim to prevent a progressive decline of ventricular function.

Fibroblast activation protein (FAP), a marker for active fibroblasts, has been observed to be highly expressed in myofibroblasts in the hearts of rats with permanent myocardial infarction (MI) and in patients with acute MI (7). In vitro, FAP was induced by TGF-β_1_ via the canonical SMAD2/SMAD3 pathway (7). Radiolabeled FAP inhibitors (FAPIs) for noninvasive imaging of FAP expression have been reported by Linder's group, used for diagnosis and treatment of tumor patients (8). Furthermore, in a small sample study by Diekmann et al. (*n* = 34), the non-invasive imaging method using ^68^Ga-FAPI-04 PET/CT has shown feasibility in assessing activated fibroblasts in patients with MI following early reperfusion therapy and predicting the progression of contractile dysfunction (9). However, data on activated fibroblasts in patients with short-term myocardial ischemia and follow-up information on those with ischemic myocardial damage are currently lacking.

This case report demonstrates the feasibility of using ^68^Ga-FAPI-04 PET/CT examination to assess early activated fibroblasts in a patient with UA and provides follow-up information.

## Case presentation

A 49-year-old male was admitted with recurrent post-exertional chest pain. The pain occurred behind the sternum during brisk walking or stair climbing, occasionally radiating to the left shoulder, lasting a few minutes, and was relieved by rest. There was no persistent chest pain exceeding 30 min. The patient had a smoking history of 30 pack-years. He was diagnosed with hypertension 5 years ago and remained untreated until starting nifedipine controlled-release tablets at 30 mg daily when blood pressure was above 200/90 mmHg; it was then maintained around 150/90 mmHg. Furthermore, he was diagnosed with primary hyperthyroidism 20 years ago and treated with radioactive iodine (I^131^). However, his thyroid hormone levels were not rechecked.

**Figure 1:**
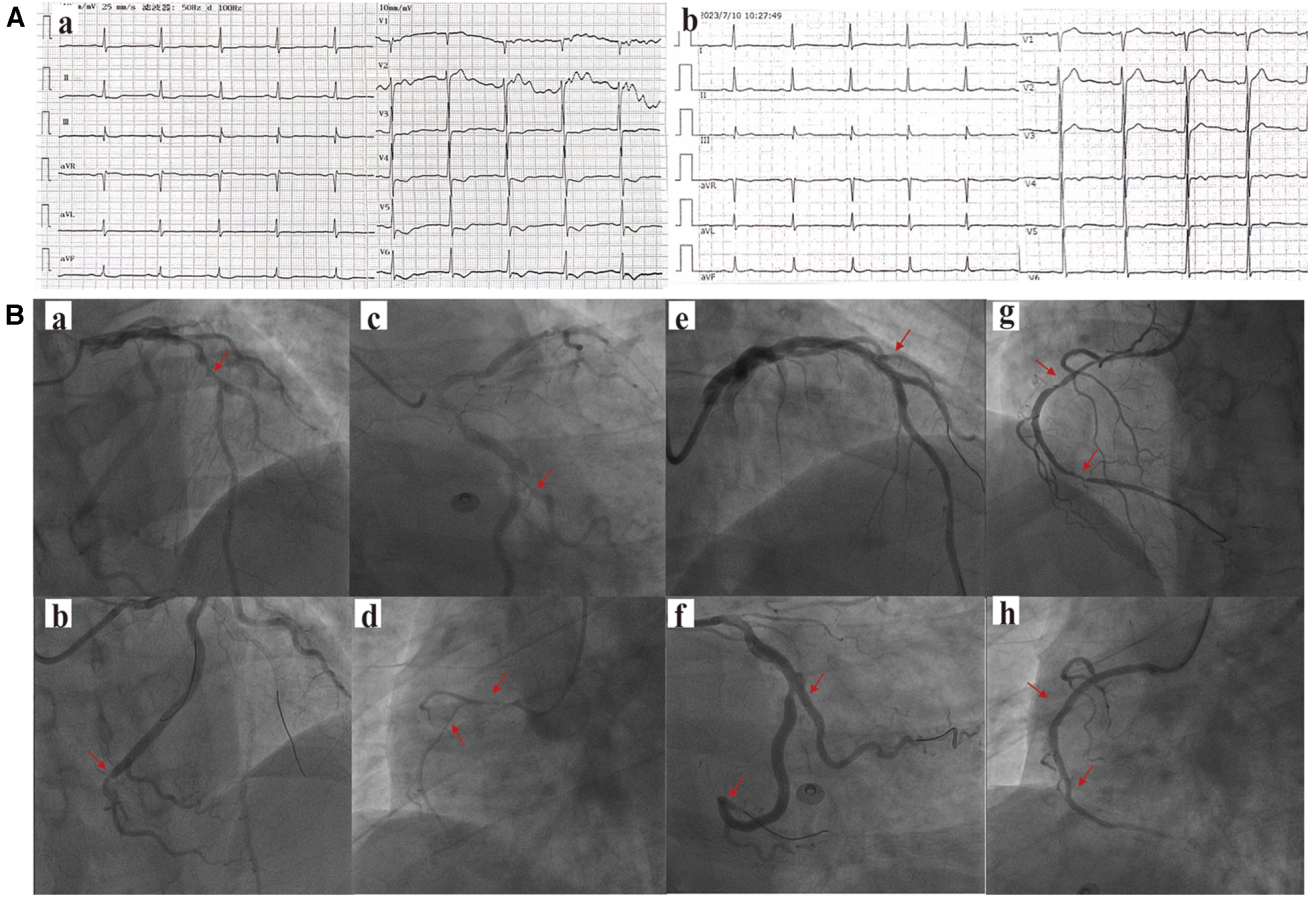
(**A**) The patient's (**a**) electrocardiogram at the time of hospital admission and (**b**) the electrocardiogram during the follow-up examination two months later. (**B**) The results of the patient's first coronary angiography are as follows: (**a**) The left anterior descending artery (LAD) mid-segment with approximately 95% stenosis, (**b**) The left circumflex artery (LCX) mid-distal segment with approximately 70% stenosis, (**c**) The dominant second obtuse marginal branch (OM2) proximal segment with approximately 90% stenosis, and (**d**) The right coronary artery (RCA) mid-segment with approximately 90% stenosis. The patient underwent the first-stage percutaneous coronary intervention (PCI) in the (**e**) LAD, (**f**) LCX, and OM2. A second-stage PCI was performed 7 days later, involving (**g**) the RCA mid-segment with approximately 90% stenosis, and (**h**) stents were implanted. The areas indicated by the arrows in the figure represent vascular stenosis or locations where stents have been implanted.

Physical examination was normal except for high blood pressure (156/91 mmHg) and obesity (BMI = 34.37 kg/m^2^). The results of the laboratory tests revealed that the patient has mild mix dyslipidemia (low-density lipoprotein 4.34 mmol/L, normal range:1–3.37 mmol/L; high-density lipoprotein 1.41 mmol/L, normal range:1.04–2.08 mmol/L; total cholesterol 7.64 mmol/L, normal range:2.9–5.18 mmol/L; triglycerides 3.66 mmol/L, normal range:0.4–1.7 mmol/L). Biochemical markers of myocardial injury included high-sensitivity troponin T (hs-TnT) mildly elevated (0.02 ng/ml, normal range <0.014), along with normal levels of creatine kinase isoenzymes (CK-MB) and myoglobin. Blood counts, serum electrolytes, fasting plasma glucose, glycated hemoglobin, liver and renal function tests, viral markers, a coagulation profile and were within normal limits. The electrocardiogram revealed ST-segment depression exceeding 0.05mv in leads II, V3-V6, accompanied by T-wave inversion (Figure 1A). Echocardiography indicated normal left ventricular systolic function but a slight decline in diastolic function with left atrial enlargement (Table 1).

**Table 1:** Two-dimensional data from the echocardiogram.

	Upon admission	2 months later
LA (mm)	44	39
LVDd (mm)	43	52
LVDs (mm)	28	35
IVS (mm)	12	12
LVPW (mm)	10	12
RA (mm)	47 × 35	40 × 27
RV (mm)	21	24
LVEF (%)	65	62
E/e’	9.88	20

LA, left atrial diameter; LVDd, left ventricular end-diastolic diameter; LVDs, left ventricular end-diastolic diameter; IVS, interventricular septum; LVPW, left ventricular posterior wall at end-diastole; RA, suitable atrium diameter; RV, right ventricular diameter; LVEF, left ventricular ejection fraction; E/e’, ratio of the early transmitral blood flow velocity to early diastolic velocity of the mitral annulus.

Other laboratory investigations revealed decreased T3 (1.10 pg/ml, normal range: 1.8–3.8 pg/ml), decreased T4 (<0.1 ng/dl, normal range: 0.78–1.86 ng/dl), and elevated TSH (56.669 mIU/L, normal range: 0.38–5.57 mIU/L), accompanied by an increased level of thyroid peroxidase antibody. These findings suggest post-radioiodine hypothyroidism in the patient.

He underwent elective coronary angiography, which unveiled multi-vessel coronary artery stenosis by physician visual assessment (Figure 1B). The SYNTAX (Synergy Between Percutaneous Coronary Intervention with Taxus and Cardiac Surgery) score was 15, indicating a moderate level of complexity in the coronary artery disease. Subsequently, the patient agreed to undergo the first stage percutaneous coronary intervention (PCI) in the LAD and OM2. A second-stage PCI for the RCA was performed 7 days later (Figure 1B). The post-procedure hs-TnT measured 0.018 ng/ml, with normal CK-MB and myoglobin, and did not significantly change during the admission.

**Figure 2:**
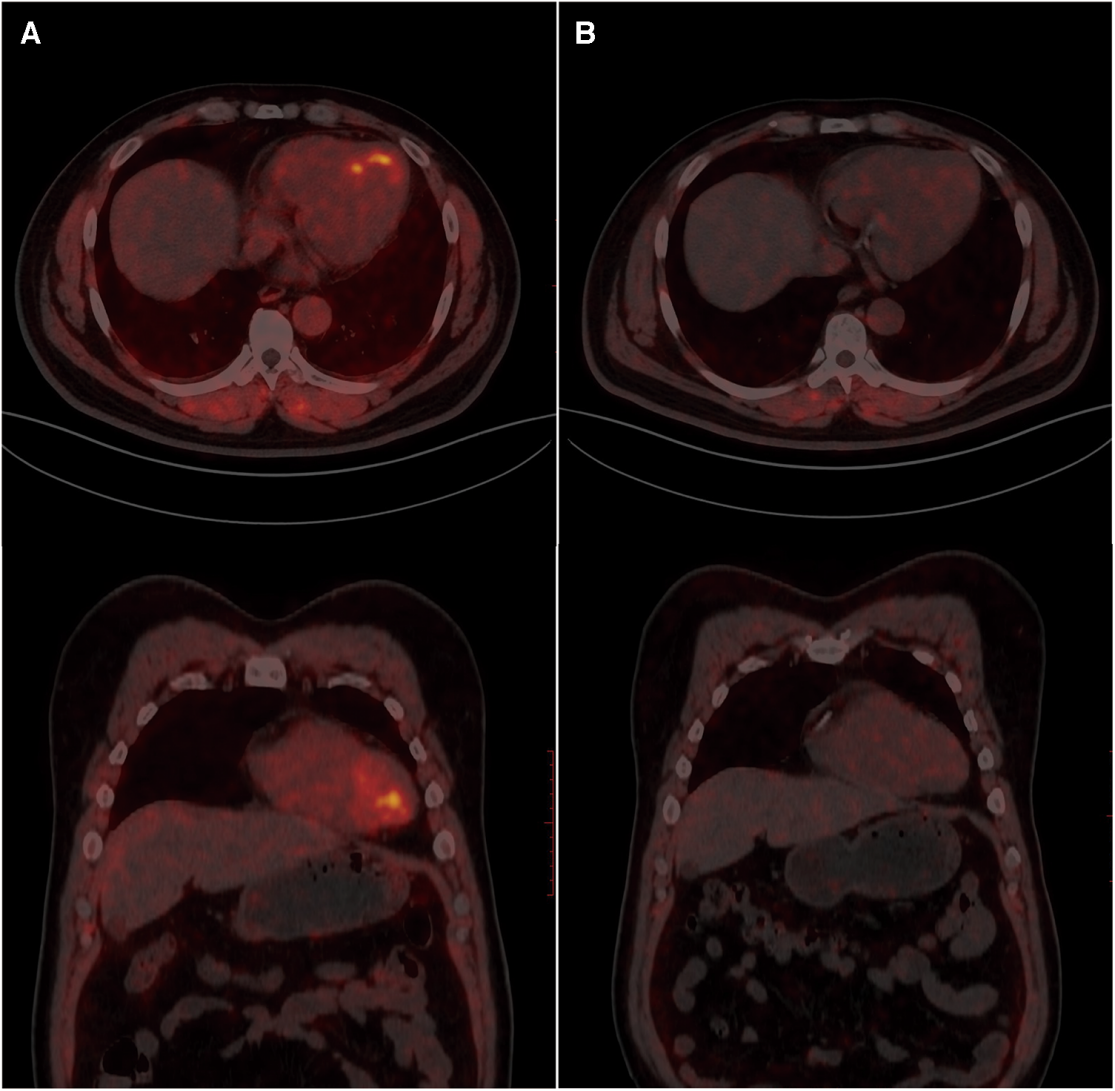
^68^Ga-FAPI-04 PET/CT myocardial imaging. A. Examination results one day after postoperative PCI. B. Follow-up examination results after two months.

To evaluate the patient's myocardial fibrosis, a ^68^Ga-FAPI-04 PET/CT examination was conducted the day following the first stage PCI (Figure 2A). The results revealed elevated FAPI uptake in the left ventricular myocardium, specifically in the apex, anterior wall, and septum, with an SUVmax (maximum standard uptake value) of approximately 4.6, consistent with the distribution of the LAD. Considering the link between ischemia and active fibroblast in pathophysiology, we hypothesize that the LAD is more likely to be the “culprit vessel” during the patient's angina attacks, while the RCA and LCX may be considered as “bystanders.” Subsequently, the patient daily received aspirin 100 mg and ticagrelor 180 mg for antiplatelet aggregation therapy, atorvastatin 20 mg and hybutimibe 10 mg for hypolipidemic therapy, sacubitril/valsartan 100 mg for antihypertensive therapy, and levothyroxine sodium tablets 50 μg for thyroid hormone supplementation. During follow-up, the patient remained free of chest pain, adhered to medication, and maintained blood pressure within the range of 120–130/70–80 mmHg.

Two months later, the patient's ECG was normal (Figure 1A), cardiac injury markers and thyroid function were normal, and there was some improvement in dyslipidemia (low-density lipoprotein 1.36 mmol/L, high-density lipoprotein 1.06 mmol/L, total cholesterol 2.87 mmol/L, triglycerides 2.15 mmol/L). A follow-up ^68^Ga-FAPI PET/CT examination (Figure 2B) revealed a slight increase in FAP expression in a smaller localized area of the left ventricular apex, with an SUVmax of approximately 1.8. The rest of the myocardium exhibited uniform tracer uptake similar to blood pool activity. Compared to the initial examination, the extent of ^68^Ga-FAPI04 uptake in the left ventricular myocardium significantly decreased, and the SUVmax notably reduced. Meanwhile, echocardiographic measurements showed mild enlargement of the left ventricle within the normal range, with unaffected systolic function (Table 1).

## Discussion

The radiotracer ^68^Ga-FAPI-04 can selectively target FAP and visualize activated fibroblasts. In this case, the left ventricular myocardium displayed focal uptake of ^68^Ga-FAPI-04 (SUVmax = 4.6), indicating the detection of active fibroblasts caused by transient myocardial ischemia was sensitive. Compared to previous studies on MI, the myocardial uptake in the UA patient was lower, potentially indicating a correlation with the extent of myocardial damage (10).

After a 2-month follow-up, a repeat ^68^Ga-FAPI-04 PET/CT revealed a significant decrease in tracer uptake in the previously affected area, without new uptake sites. The echocardiogram revealed mild left ventricular enlargement at the same time. Notably, healthy myocardium and mature myocardial scars, lacking active fibroblasts, do not exhibit uptake of ^68^Ga-FAPI-04 (11). This suggests that ^68^Ga-FAPI-04 PET/CT can provide early information on myocardial fibrosis, and has a certain predictive effect on ventricular remodeling, serving as a supplementary tool to traditional exams. On the other hand, ^68^Ga-FAPI-04 PET/CT provides insights into treatment efficacy by assessing activated fibroblasts. The patient underwent interventional therapy to address vascular narrowing and received pharmaceutical intervention for thrombosis prevention and the management of coronary risk factors, including hyperlipidemia, hypertension, andhypothyroidism. Upon reevaluation, the absence of newly activated fibroblasts suggested no recent myocardial ischemic damage, further supporting the treatment's effectiveness. In contrast, according to another study, it has been observed that a single case showed detectable high expression of FAP even 2 months after acute myocardial infarction. This difference may be associated with the distinct disease stages that the patients are in (acute phase and relatively stable phase) as well as the complete relief of myocardial ischemia (12). Therefore, more extensive cohort studies are warranted to explore further the degree, development, and outcome of myocardial fibrosis activated by different degrees of myocardial ischemia.

Furthermore, we observed concentrated ^68^Ga-FAPI-04 uptake corresponding to the myocardial ischemia territory supplied by the LAD in this case. Historically, myocardial perfusion imaging was employed to assess myocardial ischemia by observing myocardial blood flow distribution. However, it was susceptible to physiological parameters such as heart rate during the examination. The imaging process was time-consuming, required pharmacologic stress, and was unsuitable for patients in danger (13). In contrast, the examination protocol for ^68^Ga-FAPI-04 PET/CT is safer, being conducted an hour post-tracer injection (14). When referring to other molecular imaging, such as ^18^F-FDG PET, determines viability of myocytes by visualizing uptake of radiolabelled glucose analogue, rather than fibrotic tissue in imaging (6). The ^68^Ga-FAPI-04 PET/CT can not only conveniently provide areas of myocardial ischemia, but also provide fibrosis information, offering a potentially valuable means of detecting myocardial damage for patients who cannot tolerate traditional examinations and those with asymptomatic coronary artery disease.

## Conclusion

We have demonstrated the feasibility of employing ^68^Ga-FAPI-04 PET/CT to assess early myocardial fibrosis and pinpoint affected myocardium in patients with unstable angina (UA). FAP-targeted imaging holds promise as a novel biomarker for ventricular remodeling, complementing existing techniques. Moreover, it offers potential guidance for future studies on anti-fibrotic interventions.

## Data Availability

The original contributions presented in the study are included in the article/Supplementary Material, further inquiries can be directed to the corresponding author.
